# The Financial Risks of Unpaid Caregiving During the COVID-19 Pandemic: Results From a Self-reported Survey in a Canadian Jurisdiction

**DOI:** 10.1177/11786329221144889

**Published:** 2023-01-07

**Authors:** Husayn Marani, Sara Allin, Sandra McKay, Gregory P. Marchildon

**Affiliations:** 1 Institute of Health Policy, Management and Evaluation, Dalla Lana School of Public Health, University of Toronto, Toronto, ON, Canada; 2 North American Observatory on Health Systems and Policies, University of Toronto, Toronto, ON, Canada; 3 VHA Home HealthCare, Toronto, ON, Canada; 4 Department of Physical Therapy, University of Toronto, Toronto, ON, Canada

**Keywords:** Caregivers, home care, financial risk, COVID-19, Canada

## Abstract

As health service delivery shifts from institutions to the home, greater care responsibilities are being imposed on unpaid caregivers. However, gaps remain concerning how these responsibilities are contributing to caregivers’ financial risk. This study describes results from an online survey conducted in late-2020 in Ontario, Canada, about the financial risks of unpaid, homebased caregiving throughout the first year of the COVID-19 pandemic. Among 190 caregivers, salient findings include difficulties paying for care expenses after the pandemic was declared than before (*P* = .002); more caregivers retiring or becoming unemployed during the pandemic than before (*P* = .013); and a significant relationship between paying out-of-pocket for a home care worker and experiencing a decrease in the availability of such support during the pandemic (*P* = .029). Overall, the financial stressors of caregiving during the pandemic contributed negatively to caregivers’ mental health, with 64.2% noting could be partly offset by greater government and employment-based assistance in managing care expenses and productivity losses. Findings from this study will better inform policies that aim to protect unpaid caregivers from financial risk in pandemic recovery efforts and beyond. Results may also be useful in other welfare states where unpaid caregivers provide the majority of home care services.

## Introduction

Across OECD countries, demand for long-term care services is rising, in part driven by an aging population.^1^ Concurrently, we are seeing a shift in the pattern of long-term care delivery from institutions (eg, residential long-term care facilities or nursing homes) to the home. To meet this growing demand, 13.5 million home care workers will be needed by 2040 to fill care deficits in long-term care provision, which is putting immense pressure on unpaid caregivers to fill these gaps.^1^ Due to the informal nature of care they provide and diversity in care activities performed, obtaining comparable cross-country data on the number of unpaid caregivers and frequency of caregiving is challenging, but it is estimated that 13% of people over 50 provide weekly unpaid care across OECD countries.^2^ Much of this care is and will continue to be provided within the home.^3^

Similar patterns exist in Canada, where 96% of persons receiving care at home (“home care”) have an unpaid caregiver.^4^ Gaps in the supply of home care provided by a home care worker (see operational definition in Table 1) have been filled by unpaid caregivers, who have taken on more weekly hours providing unpaid care.^4^ Against the backdrop of the COVID-19 pandemic, which, in Canada, ravaged residential long-term care facilities,^5^ and a growing desire to receive care at home,^6^ the sustainability of unpaid caregiving in the provision of home care is becoming an increasingly important area of health policy inquiry in Canada.

**Table 1.** Operational definitions of key home care terms used in this analysis.

Term	Operational definitions of key home care terms used in dissertation
Home care	The provision of full- or part-time care, including social, psychological, emotional, and physical attention, for any duration of time (short- or long-term basis) for people living in their home or the home of a relative or friend. Home care provision may be paid or unpaid (depending on political choices, cultural beliefs, and gender structures) on the basis of a voluntary or professional agreement, or moral obligation.
Long-term care (services)	Services delivered or tasks/activities performed that help meet the medical and non-medical care needs of individuals living with a health condition, injury, or age-related decline. Non-medical activities include support for activities of daily living including grooming, feeding, dressing, bathing, toileting and transferring. Such activities may be performed by a home care worker (specifically a personal support worker in the Ontario context) or an unpaid caregiver. Long-term care may be provided in an institution, usually a residential long-term care facility and sometimes a hospital, or at home (including an individual’s home or a private retirement home). While long-term care can be provided on a long-term basis (greater than 3 months), it may also be provided on a short-term basis (less than 3 months).
Residential long-term care facility	An institution (facility) in which long-term care is provided for persons who cannot take care of themselves on their own (independently).
Home care worker (personal support workers in Ontario)	A preferred term for those who receive compensation for the care activities they perform for those living at home or at an institution (eg, residential long-term care facility).^7^ In Ontario, the term “home care worker” is increasingly synonymous with personal support workers (“PSWs”), whose scope of practice involves supporting activities of daily living. PSWs may be employed privately by an unpaid caregiver/care recipient who pays out-of-pocket, or by a service provider organization (“SPO”). If the latter, PSWs may be allocated to care recipients through a regional health authority (eg, Home and Community Support Services in Ontario) that contracts SPO-employed PSWs, or directly by an SPO. In both cases, PSW hours are government-subsidized to an extent and needs-tested.^8^
Unpaid Caregiver	A family member, friend, or neighbor who provides care on fully a voluntary (unpaid) basis for someone needing support with activities with daily living (grooming, feeding, dressing, bathing, toileting, and transferring) or instrumental activities of daily living (meal preparation, medication management, banking, and financing, transportation to and from medical appointments, indoor and outdoor household activities (laundry, maintenance, and technology use) due to a health condition, injury, or age-related decline.
Direct Costs	Any medical or non-medical expense associated with the care of an individual. May be incurred by public (government), or privately (through insurance or out-of-pocket by a patient (care recipient) or their caregiver).
Out-of-Pocket Cost	Any expense (eg, related to caregiving) paid for directly by individuals and not fully or partially reimbursed.
Reimbursement	A payment made by a third party (eg, insurance provider or government) to a patient (care recipient) or caregiver for a product or service.
Indirect Costs	Quantifiable costs not directly related to medical and non-medical care, including lost wages from taking time off work to provide care and the monetary value of time spent caregiving (unpaid labor).
Financial Risk	The consequences of direct, private (out-of-pocket) health care expenditure, including both the magnitude of out-of-pocket expenditure, and the impacts of these expenses across domains of financial risk, including income-generating potential, employment productivity, and overall personal health. Protecting individuals from financial risk is a critical element of universal health coverage in welfare states.

One reason the sustainability of unpaid caregiving is gaining policy attention is because of the corresponding financial challenges, or risks, caregivers may experience across domains such as income level, employment productivity, and personal health. These risks could be associated with the direct costs of equipment and care supplies incurred by caregivers, and indirect costs from taking time off work to provide care due, in part, to gaps in the availability of home care workers.^9,10^ There is also a growing concern that caregivers will become costly users of the health system as a result of stressors, financial and otherwise, associated with unpaid caregiving.^11,12^ This concern has been exacerbated by stressors associated with the pandemic, which have been experienced disproportionately by women caregivers.^13^

## Financial Risk Protection and Unpaid Caregiving

Mitigating the direct and indirect costs of caregiving could address some financial risks of unpaid caregiving. In general, financial risk protection is a primary goal of welfare states, including Canada, in an effort to stimulate employment productivity, income-generating potential, and personal health.^14^ In traditional conceptions of the welfare state, protecting individuals from financial risk may involve direct income support, including subsidies and cash transfers.^15^ Broadening this notion in the context of health care, one of the main policy levers to achieve financial risk protection is publicly subsidized/financed health coverage, which aims to mitigate the risks associated with private (out-of-pocket) health care expenditure.^14,16,17^

Evidence of financial risk across health sectors (including home care) may be indicative of gaps in existing approaches to financial risk protection. As demand for home care services increases,^6^ debates are emerging concerning the expansion of universal health care in Canada (Medicare) to include an array of otherwise excluded extended home care services.^18^ This has important implications on unpaid caregivers, who are often proxy health care decision-makers for their care recipient(s), and may accept responsibility for care-related expenses.^19^ Further, the landscape of home care is changing. Events such as the COVID-19 pandemic, coupled with ongoing trends like the rapidly rising rate of dementia, a constrained health care budget, challenges recruiting and sustaining health human resources, and a shift of costs onto individuals and families as care moves out of institutions and into home and community,^20-22^ call into question the extent to which existing approaches that protect unpaid caregivers from the financial risks of caregiving reflect the current conditions under which they now operate.

At the same time, policy actions to address the financial risks of unpaid caregiving are constrained by evidence gaps concerning the current state of financial risk in Canada. The paucity of research in this area could be due to challenges in being able to accurately capture self-reported private care expenditure.^23^ A recent document review of federal surveys administered by Statistics Canada found that questions about the financial aspects of caregiving were largely derived from 2 surveys, the General Social Survey and the Canadian Community Health Survey.^24^ However, gaps exist concerning questions related to estimates of private care expenditure, and the impacts of caregiving across various domains of financial risk.^24^

The paucity of research on the financial risks of unpaid caregiving could also be attributed to the way private (out-of-pocket) health care expenditure and private insurance is represented in government administrative datasets such as the National Health Expenditure Database (NHEX). With the exception of pharmaceuticals, vision and dental expenditure, private health care expenditure—which makes up 30% of total national health expenditure in Canada—related to home care (eg, supplemental support from a home care worker and assistive medical devices) are not recorded in NHEX.^25^ This means we are unable to identify where private expenses come from. It also means we may be underestimating cumulative private expenditure from all sources. Even with data on out-of-pocket expenditure related to caregiving, it is still challenging to measure financial risk associated with indirect costs such as time taken off work to provide care. These gaps hide the magnitude of financial challenges among unpaid caregivers. Accordingly, strategies that attempt to offset the financial risks of caregiving are informed by information that does not present a fulsome picture of the range, nature and extent of the financial burden of caregiving on unpaid caregivers.

Thus, in this paper, we sought to examine the financial risks of unpaid, homebased caregiving in 1 Canadian province (Ontario). Our analysis uses data from an original, self-reported, cross-sectional survey developed by the study team—the methods of which have been described elsewhere^26^—to describe the magnitude of unpaid care expenditure, and how this impacts caregivers across domains of financial risk, including income-generating potential, employment productivity, and overall personal health. Results from this study provide a more fulsome understanding costs of unpaid caregiving, particularly during the COVID-19 pandemic. Accordingly, results may better inform short- and long-term approaches that protect unpaid caregivers from the financial risks of caregiving.

## Methods

### Survey development and eligibility

There is no single, validated survey in the literature that explores the financial risks of unpaid, homebased caregiving. As such, we developed an online, cross-sectional survey, the methods for which have been published elsewhere.^26^ In short, this survey consolidates questions from existing, validated survey instruments in the literature, as well relevant, but limited, questions from existing public (government-sponsored) surveys, including the General Social Survey (GSS) and the Canadian Community Health Survey (CCHS). We filled remaining gaps with original questions that elucidate the impact of the pandemic on caregivers’ care-related expenditure and associated financial risks; for example, “Has COVID-19 affected your employment status [yes or no]”; “Since the pandemic was declared, have your weekly care expenses increased, decreased or stayed the same?”; and “As a result of the COVID-19 pandemic, have the number of hours of home care you receive from a personal support worker increased, decreased or stayed the same?”^26^ Although some questions were derived from existing Canadian surveys, the language of survey items is not specific to any jurisdiction, and response options are adaptable to fit other contexts. For example, response options concerning sources of income include several provincial and federal benefits, which can be excluded or revised should this survey be conducted in other settings.^26^

The survey was field tested in a consultative process with 10 homebased caregivers. Principal feedback concerned the use of lay language and a shorter recall period (3 months) for cost estimates to ensure accurate estimates and reduce cognitive strain on caregiver participants.^26^ The final composite survey consists of 72 items (questions) organized into 6 sections: (a) demographics of caregiver; (b) profile of care recipient; (c) direct costs of caregiving; (d) caregivers’ personal income and household income sources; (e) caregiver employment; and (f) caregiver health and quality of life. Given the sensitive and personal nature of questions related to income, employment and costs of caregiving, the survey is voluntary, anonymous and confidential survey.^26^

This study presents results from the first application of this survey in Ontario, Canada. Ontario is home to an estimated 3.3 million unpaid caregivers, 83% of whom provide care for someone living at home.^27^ We opted to focus on Ontario because home care is an extended service in Canada, not an insured service under the *Canada Health Act* (1984). Accordingly, there is no obligation for provincial or territorial governments, which are responsible for the delivery of publicly funded health services through Canada’s system of universal health coverage (Medicare), to provide these services. Public coverage of home care devices and home care provided by a home care worker (Table 1) therefore differs across jurisdictions.^28^ Leveraging results from this pilot test in Ontario, we intend on conducting this survey across other provinces and international jurisdictions in the near future.

To participate in the survey, caregivers had to be a resident of Ontario, at least 16 years old (Ontario’s legal working age), and providing care on a voluntary basis for at least 1 person living at home, independently or otherwise, with a health condition or limitations in activities in daily living due to injury, disability or age-related decline. Further, caregivers had to have been providing care for at least 3 months (corresponding to the recall period of survey questions) and within the time period of Ontario’s COVID-19 state of emergency—formally declared in Ontario on March 17, 2020^29^ and terminated on February 8, 2021^30^—to better facilitate comparison around care-related costs.

### Recruitment

Caregivers were recruited to participate in the online survey between August and December 2020. We drew upon best practices in conducting research using the internet and engaging diverse audiences.^31,32^ A combination of active and passive (snowball) approaches were used. Specifically, a brief description of this study consisting of a link to the survey and promotional flyers were sent by members of the research team to over 100 organizations across Ontario to circulate to their caregiving networks. Organizations included patient and caregiver advisory groups across Ontario hospitals and regional health authorities (Home and Community Care Support Services, or “HCCSS”), and the Ontario Caregiver Organization to name a few. Word-of-mouth and social media were also used, including Facebook’s paid ad program and Twitter.

The survey was hosted on REDCap (Research Electronic Data Capture), a safe and secure web-based survey platform.^33^ Prior to starting the survey, caregivers read a brief description of the survey that provided all necessary information to make an informed decision about their participation. By agreeing to participate in the anonymous survey, caregivers provided their informed consent to participate. At the end of the survey, caregivers provided only their e-mail address if they were interested in receiving an honorarium for their participation.

### Analysis

As a descriptive cross-sectional survey, we aimed to reduce estimation (type 1) errors by recruiting as large a sample as possible within pandemic-related constraints, and reporting standard deviation (SD) or 95% confidence intervals (CI) around relevant population proportions and sample means. To facilitate some preliminary comparisons between categorical variables which could inform future research, we aimed to meet a frequency of at least 5 observations per response option in each categorical variable.^34^ We do not report any statistical analysis (descriptive or otherwise) where this benchmark is not met.

Results were analyzed descriptively using IBM SPSS Statistics V26.^35^ First, we summarized the sociodemographic profile of caregivers and their care recipients to contextualize our results. We then described the main categories of care-related expenses, and the magnitude and impacts of care expenditure across the domains of financial risk.

Given the small sample size relative to the number of variables, and the possibility of missing responses that would compromise normal distribution, any statistical tests we used were largely non-parametric. For tests involving paired matching of nominal variables (eg, ability to meet care expenses and employment status before and during the pandemic), we utilized McNemar (binomial) tests; for tests of independence of 2 or more categorical groups, we utilized Mann-Whitney U or Kruskal Wallis H test, respectively; and for cross-tabulation of categorical variables, we utilized Pearson chi-squared (*χ*^2^) tests. In all tests, *P* < .05. Where relevant, tests excluded missing and “other” values. Accordingly, in our results, n refers to the number of respondents to questions asked of the whole sample (less missing and “other” variables if applicable), and ns refers to the number of respondents in subsample questions.

As all cost estimates were collected from the previous 3 months using ordinal, self-reported cost ranges, we did not adjust any costs in accordance with the current consumer price index. To facilitate descriptive analysis, the mid-point of cost ranges was then derived, and outliers with an absolute *z*-score value of ±3.29 were removed.^36^ All costs are reported in 2021 Canadian dollars where $1 CAD = $0.79 USD as of August 19, 2021.

This study was approved by the Research Ethics Board at a large university in Ontario, Canada.

## Results

In total, 190 caregivers participated in this survey. Given the various modes of survey recruitment, estimating a response rate based on recruitment approaches was not possible. Accordingly, the maximum margin of error (MOE) for our sample was ±6% at a 95% level of confidence based on a liberal estimate (3.3 million) of caregivers in Ontario.^27^

### Profile of caregivers and care recipients

The average age of caregivers was 57.8 (range 24-93; SD 12.6; 95% CI 55.9-59.6), 87.8% were women, and 82.6% were born in Canada. Additional demographic data can be found in Table 2.

**Table 2.** Sociodemographic characteristics of caregivers (n = 190).

Variable		n	% of total sample	% of total sample excluding missing/other	95% CI, LB %; UB %
Age	⩽30	5	2.6	2.2	0.9; 6
	31-40	16	8.4	8.6	4.9; 13.3
	41-50	33	17.4	17.7	12.3; 23.5
	51-60	51	26.8	27.4	20.7; 33.7
	61-70	53	27.9	28.5	21.6; 34.8
	71-80	25	13.2	13.4	8.7; 18.8
	⩾81	4	2.1	2.2	0.6; 5.3
	Missing^a^	3	1.6	N/A	-
Gender	Man (Boy)	22	11.6	11.7	7.4; 17.1
	Woman (Girl)	166	87.4	88.3	82.3; 92.1
	Other gender identity	1	0.5	N/A	-
	Missing^a^	1	0.5	N/A	-
Marital Status	Married	99	52.1	52.9	45.3; 60
	Common law	19	10	10.2	6.2; 15.3
	Widowed	18	9.5	9.6	5.8; 14.7
	Separated or Divorced	18	9.4	9.6	5.8; 14.7
	Single	33	17.4	17.6	12.4; 23.8
	Other	1	0.5	N/A	-
	Missing^a^	2	1	N/A	-
Birthplace	Within Canada	157	82.6	84.9	76.5; 87.7
	Outside Canada	28	14.7	16.1	10.0; 20.6
	Missing^a^	5	2.6	N/A	-
Race^b^	Black	2	1.1	1.1	0.1; 3.9
	East Asian	5	2.6	2.7	0.9; 6.2
	Latino	3	1.6	1.6	0.3; 4.7
	Middle Eastern	3	1.6	1.6	0.3; 4.7
	South Asian	8	4.2	4.4	1.9; 8.3
	White of European Descent	154	81.1	84.2	77.1; 88.3
	Mixed	8	4.2	4.4	1.9; 8.3
	Missing^a^	7	3.7	N/A	-
Postal district^c^	K (Eastern Ontario)	49	25.8	25.9	19.7; 32.6
	L (Southwestern Ontario)	57	30	30.2	24.1; 37.6
	M (Central Ontario)	49	25.8	25.9	19.7; 32.6
	N (Southern Ontario)	29	15.3	15.3	10; 20.6
	P (Northern Ontario)	5	2.6	2.6	0.9; 6
	Missing^a^	1	0.5	N/A	-
Employment Status	Working, Self-employed	25	13.2	14.5	8.8; 19.1
	Working, Employed	61	32.1	35.5	26; 39.8
	Not working (Retired)	67	35.3	39.0	29; 43.2
	Not working (Will return later)	19	10.0	11.0	6.2; 15.4
	Other^d^	10	5.3	N/A	-
	Missing^a^	8	4.2	N/A	-
Gross personal income^e^ (previous 12 months)^f^	Less than $10 000	11	5.8	6.5	3; 10.3
	$10 001-$30 000	44	23.2	26.2	17.7; 30.4
	$30 001-$50 000	38	20	22.6	14.9; 26.9
	$50 001-$70 000	28	14.7	16.7	10.2; 21
	$70 001-$90 000	19	10	11.3	6.3; 15.5
	$90 001+	28	14.7	16.7	10.2; 21
	Missing^a^	22	11.6	N/A	-

a Missing values due to selection of “don’t know,” “prefer not to answer,” or caregiver skipped this question. Missing responses excluded from analysis or treated as “not yes” where applicable.

b Categories informed by standards and guidance from the Government of Ontario Anti-Racism Directorate.

c Ontario postal districts separate Ontario into 5 broad urban and rural regions.^37^

d Free-text responses: on long-term disability (6), parental leave (1), caregiver (2), volunteer (1), student (2).

e Represents individual earnings (wages and salaries) as well as contributions from other sources, including social security programs, interest, dividends, employment insurance, retirement savings (Supplemental Figure 2).

f All values are in Canadian dollars ($1 CAD = $0.79 USD as of August 2021).

Most caregivers (74.7%; 95% CI 67.9%-80.7%) provided care for one dependent person, mainly their parent (40.5%; 95% CI 33.5%-47.9%), offspring (24.2%; 95% CI 18.3%-30.9%), or spouse (21.6%; 95% CI 16%-28.1%). The most commonly reported care activities included (1) transportation to and from medical appointments (90%; 95% CI 84.8%-93.9%), (2) communication with health and medical professionals (88.4%; 95% CI 83%-92.6%), (3) indoor household chores (84.7%; 95% CI 78.8%-89.5%), and (4) banking/financing (81.1%; 95% CI 74.7%-86.4%).

Care recipients had a number of health conditions (Table 3), most notably frailty as a result of aging, dementia, urinary or bowel incontinence, and arthritis. Across all care provided, caregivers provided on average 51.7 (range 2-168; SD 51.033; 95% CI 43.6-58.3) hours of unpaid care per week. A Mann-Whitney U test indicated that weekly hours of care performed was significantly higher for care recipients with the following health conditions relative to those without: frailty due to aging (*U* = 3486; *P* = .049; df = 1), an intellectual or developmental disability (*U* = 1442.5; *P* = .001; df = 1), asthma (*U* = 464.5; *P* = .035; df = 1), and urinary or bowel incontinence (*U* = 1579.5; *P* = .016; df = 1).

**Table 3.** Care recipients’ health conditions.

Distribution of health conditions^a^ (n = 187)	n	% of sample	95% CI LB%; UB%
Aging or frailty	78	41.1	34; 48.4
All other neurological conditions	39	20.5	15; 27
Alzheimer’s disease or related dementia	65	34.2	27.5; 41.4
Arthritis	48	25.3	19.3; 32.1
Asthma	9	4.7	2.2; 8.8
Back problems	27	14.2	9.6; 20
Cancer	16	8.4	4.9; 13.3
Cardiovascular disease	35	18.4	13.2; 24.7
Chronic bronchitis, emphysema, COPD	16	8.4	4.9; 13.3
Intellectual or developmental disability	31	16.3	11.4; 22.4
Diabetes	30	15.8	10.9; 21.8
Digestive conditions	12	6.3	3.3; 10.8
Fibromyalgia or chronic fatigue	9	4.7	2.2; 8.8
Injury from accident	8	4.2	1.8; 8.1
Kidney disease	14	7.4	4.1; 12.1
Mental health condition	40	21.1	15.5; 27.5
Migraine	4	2.1	0.6; 5.3
Osteoporposis	20	10.5	6.5; 15.8
Urinary or bowel incontinence	49	25.8	19.7; 32.6
Other (free-text response)	36	18.9	13.6; 25.3

a List derived from the Canadian Community Health Survey.^38^

Nearly half (47.4%) of all caregivers (n = 90; 95% CI 40%-55%) did report receiving supplemental, non-medical support from a home care worker obtained publicly through HCCSS (ns = 65; 72.2%; 95% CI 61.8%-81.1%), a service provider organization (ns = 30; 33.3%; 95% CI 23.7%-44.1%), or privately through a social media posting or family/friend referral (ns = 12; 13.3%; 95% CI 7.1%-22.1%). 38.9% of caregivers receiving support from a home care worker (ns = 35; 95% CI 29.1%-50.3%) noted a decrease in availability (weekly hours worked) during the pandemic (past 3 months).

Albeit a small to medium effect size (φ), significant relationships were observed between receipt of home care from a home care worker and care recipient experiencing frailty due to aging (χ^2^ = 7.15; *P* = .007; df = 1; φ = 0.19), any neurological condition (χ^2^ = 3.95; *P* = .047; df = 1; φ = 0.14), and Alzheimer’s disease or related dementia (χ^2^ = 11.79; *P* = .001; df = 1; φ = 0.25).

### Characterizing care expenditure

Table 4 summarizes the types, frequency and magnitude of care-related direct costs. Caregivers reported incurring an expense across an average of 7 types of costs (SD 3.79; 95% CI 6.45-7.53) over their caregiving history. In the previous 3 months specifically, caregivers incurred expenses across fewer expense categories (mean 3.6; SD 3.52; 95% CI 3.12-4.11), most commonly for transportation for their care recipient (gas or ride-sharing), over-the-counter medications, and COVID-19 personal protective equipment (Table 4).

**Table 4.** Overview of types, frequency and magnitude of care-related costs.

Cost Type	Definition	Historical (n = 190)	Past 3 months^f^ (n = 124)^d^				Magnitude of Expense		
		N (%) [95% CI LB; UB]	n(%)[95% CI LB; UB]	Paid fully OOP (%^a^) [95% CI LB; UB]	Paid partly OOP^b^ (%^a^) [95% CI LB; UB]	Both^c^ (%^a^)[95% CI LB; UB]	Ns (%^a^ response rate)	$ Min; Max	$ Mean (SD) [95% CI LB; UB]
(a) Interior/ exterior home renovations	Interior/exterior home upgrades, adaptations, repairs, maintenance, renovations to improve functionality and accessibility for care recipient (eg, entrance ramp, bathroom grab bars, wider doorways, lower counters, etc.)	110 (57.9) [0.508; 0.649]	49 (25.8) [0.197; 0.326]	30 (68.2) [0.524; 0.814]	8 (18.2) [0.082; 0.327]	6 (13.6) [0.052; 0.274]	42 (85.7)	125; 27 500	2770.8 (5864.5) [943.3; 4598.3]
(b) Digital, virtual or artificial health technologies	Digital, virtual or artificial health technology (eg, smart home monitoring technology, sensors, wearable devices such as a watch or clip-on device, video cameras, therapeutic robots, electronic tablets, Life/Medic Alert, etc.)	79 (41.6) [0.345; 0.487]	38 (20) [0.146; 0.264]	19 (55.9) [0.379; 0.728]	6 (17.6) [0.068; 0.345]	9 (26.5) [0.129; 0.444]	32 (84.2)	125; 2250	496.1 (500.5) [315.7; 676.5]
(c) Medical equipment and non-digital assistive devices and mobility aides	Non-digital assistive devices and mobility aides (eg, ventilator, dialysis machine, blood pressure monitors, hearing aids, wheelchairs, three-or four-wheeled scooters, hospital beds, electronic stair lift, airway pressure machines, etc.)	99 (52.1) [0.449; 0.593]	42 (22.1) [0.164; 0.287]	17 (50) [0.324; 0.676]	8 (23.5) [0.107; 0.412]	9 (26.5) [0.129; 0.444]	34 (81.0)	125; 4750	933.8 (1190.7) [518.5; 1349.3]
(d) Personal hygiene products/ supplies	Non-COVID-related personal hygiene products (eg, diapers, catheters, dressing, etc.)	112 (58.9) [0.519; 0.660]	67 (35.3) [0.285; 0.425]	46 (71.9) [0.592; 0.824]	7 (10.9) [0.045; 0.212]	11 (17.2) [0.089; 0.287]	62 (92.5)	125; 2750	383.1 (437.6) [271.9; 494.2]
(e) COVID-19-related personal protective equipment	COVID-related personal protective equipment and supplies (eg, face masks, gloves, sanitizer, etc.)	134 (70.5) [0.640; 0.771]	88 (46.3) [0.391; 0.537]	70 (83.3) [0.736; 0.906]	8 (9.5) [0.042; 0.179]	6 (7.1) [0.027; 0.149]	84 (95.5)	125; 875	190.5 (150.9) [157.7; 223.2]
(f) Paid medical care/services (physical health)	Paid medical care/services within or outside the home setting for care recipient(s)’ physical health (eg, physiotherapy, massage therapy, occupational therapy, skin care, vision care), etc.)	102 (53.7) [0.465; 0.608]	52 (27.4) [0.212; 0.343]	19 (47.5) [0.315; 0.639]	12 (30) [0.166; 0.465]	9 (22.5) [0.108; 0.385]	39 (75.0)	125; 2250	621.8 (601.2) [426.9; 816.7]
(g) Paid medical care/services (mental, intellectual or cognitive health)	Paid medical care/service within or outside the home setting for care recipient(s) mental health (eg, psychiatrist, social worker, behavioral therapist, psychologist, etc.)	49 (25.8) [0.195; 0.321]	18 (9.5) [0.057; 0.146]	6 (54.5) [0.234; 0.833]	3 (27.3) [0.06; 0.61]	2 (18.2) [0.023; 0.518]	11 (61.1)	125; 3750	897.7 (1220.7) [77.7; 1717.8]
(h) Paid non-medical day-to-day home care provided by a home care worker	Paid, regularly occurring in-home (non-medical) support (personal support worker, home help aide, live-in paid provider, etc.) who provide support in activities of daily living (dressing, medication management, bathing, housekeeping, feeding, etc.)	48 (25.3) [0.190; 0.315]	27 (14.2) [0.096; 0.200]	8 (33.3) [0.156; 0.553]	11 (45.8) [0.256; 0.672]	5 (20.8) [0.071; 0.422]	24 (88.9)	125; 17 500	4494.8 (4679.5) [2518.8; 6470.8]
(i) Temporary institutional care	Short-term care (one or more overnight stay) at a hospital, residential long-term care facility, rehabilitation facility, etc. unrelated to respite^g^ purposes.	26 (13.7) [0.088; 0.186]	9 (4.7) [0.022; 0.088]	N/A [0; 0.602]	3 (75) [0.194; 0.994]	1 (25) [0.006; 0.806]	4 (44.4)	125; 3750	1343.8 (1634.2) [1256.6; 3944.1]
(j) Non-prescription, over-the-counter medication	Over the counter (non-prescription) drugs/medication	149 (78.4) [0.725; 0.843]	81 (42.6) [0.355; 0.500]	55 (72.4) [0.609; 0.82]	12 (15.8) [0.084; 0.26]	9 (11.8) [0.056; 0.213]	75 (92.6)	125; 2250	250 (294.2) [182.3; 317.7]
(k) Prescription medication	Prescription drugs/medication	103 (54.2) [0.471; 0.614]	57 (30) [0.236; 0.371]	24 (46.2) [0.322; 0.605]	19 (36.5) [0.236; 0.51]	9 (17.3) [0.082; 0.303]	50 (87.7)	125; 875	285 (235.6) [218; 352]
(l) Transportation or moving expenses	Transportation for your care recipient(s) to health/medical appointments and/or moving expenses to and from home (eg, driving your own vehicle (gas expense), taxi/ride-sharing costs, public transportation, renting a moving vehicle, etc.)	156 (82.1) [0.766; 0.876]	84 (44.2) [0.370; 0.516]	59 (75.6) [0.646; 0.847]	11 (14.1) [0.073; 0.238]	8 (10.3) [0.045; 0.192]	77 (91.7)	125; 4250	475.7 (732.5) [641.9; 352.2]
(m) Vehicle repairs or upgrades	Vehicle upgrades or adaptations to make it more accessible for your care recipient(s).	35 (18.4) [0.129; 0.240]	15 (7.9) [0.045; 0.127]	6 (75) [0.349; 0.968]	1 (12.5) [0.003; 0.527]	1 (12.5) [0.003; 0.527]	8 (53.3)	125; 1750	796.9 (589.9) [303.7; 1290]
(n) Recreation and leisure	Recreational activities for your care recipient(s) social well-being (eg, day programs, etc.)	85 (44.7) [0.376; 0.519]	42 (22.1) [0.164; 0.287]	23 (65.7) [0.478; 0.809]	8 (22.9) [0.104; 0.401]	4 (11.4) [0.032; 0.267]	33 (78.6)	125; 1750	439.4 (489.3) [265.9; 612.9]
(o) Other care expenses	Example, groceries, clothing	41 (21.6)^e^ [0.157; 0.275]	17 (8.9) [0.053; 0.139]	10 (90.9) [0.587; 0.998]	1 (9.1) [0.002; 0.413]	N/A [0; 0.285]	10 (58.8)	125; 32 500	4437.5 (10 104.4) [2790.7; 11 665.7]

a Based on *n* sample size from the past 3 months.

b Partially subsidized by another source (eg, private, supplemental insurance, government).

c For caregivers who incurred multiple expenses in an expense category and paid for some fully out-of-pocket and others partially out-of-pocket.

d Due to non-response, it is possible that the sum of “paid fully,” “paid partly,” and “both” does not equal the sample size (*n*) for those who incurred an expense in the past 3 months.

e Adaptive clothing (1), vehicle modification (3), footwear (2), computer tablets (2), special diet (1), dental care (1), eating out (1), educational advisor/tutor (2), groceries (6), safety (1), hospital parking (1), house cleaning (3), transport (3), paid neighbor/someone else (2), furniture (2), skills/support, allied care (1).

f All expenses incurred in the past 3 months are captured in expenses incurred historically.

g Short-term provision of companionship, personal care, or home maintenance (maid) services intended to give caregiver a break from caregiving. Can be provided in-home or in a care setting (eg, adult day care or residential long-term care facility).

In the previous 3 months, the highest expenditure was associated with supplemental, non-medical support from a home care worker, interior/exterior renovation, temporary institutional care, and medical equipment (Table 4). For each cost category, the majority of caregivers (more than half of respondents reporting incurring an expense) paid for care expenses fully out-of-pocket, except for support from home care worker and temporary institutional care. In these cases, more caregivers reported incurring a co-payment (with the remainder subsidized by another source) than paying for the expense fully out-of-pocket (Table 4).

It is worth noting that, of the caregivers who reported paying for a home care worker out-of-pocket fully or partially sometime in the past (Table 4, row h), fewer caregivers (ns = 27) paid for such support in the past 3 months specifically. This may be attributable to a change in availability of home care (hours of home care allocated) during the pandemic, as there was a significant relationship between whether or not a caregiver pays for home care from a home care worker out-of-pocket and experiencing a decrease in the availability of such support during the pandemic (χ2 = 7.12; *P* = .029; df = 2; φ = 0.28). However, there was no significant relationship between weekly hours spent caregiving and any change in the availability of support from a home care worker during the pandemic (Kruskal-Wallis χ^2^ = 3.23; *P* = .199; df = 2).

Caregivers who were not paying for support provided by a home care worker (n = 100; 52.6%; 95% CI 45.3%-59.9%) either did not require it, or reported barriers to access, including its prohibitive cost and resistance from their care recipient (Supplemental Figure 1).

### Impact of caregiving and care costs across the domains of financial risk

This section reports a detailed and descriptive analysis of how costs associated with caregiving have contributed to financial risk across domains such as including income, productivity, and personal health. Where applicable, these risks are described in relation to the ongoing COVID-19 pandemic.

#### Income

Caregivers derived gross income from an average of 2.56 sources (SD 1.76; 95% CI 2.32-2.82) in the past 3 months, the most commonly reported being wages and salaries, benefits from the Canada Pension Plan, Old Age Security, and job-related retirement pension. Few caregivers qualified for any pandemic-related federal benefits (Supplemental Figure 2).

**Figure 1.**
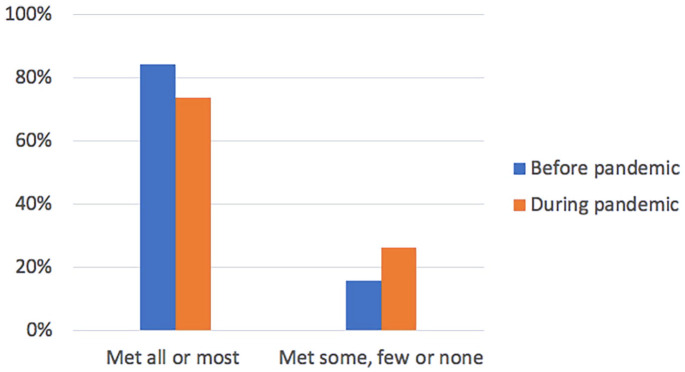
Change in ability to meet care-related expenses before & during pandemic (n = 182).

Over the previous 3 months, 32.4% of all caregivers (n = 59; 95% CI 25.7%-39.7%) reported a decrease in income and 45.6% (n = 83; 95% CI 38.2%-53.1%) reported an increase in weekly care-related out-of-pocket expenditure. A binomial test demonstrated a statistically significant difference in caregivers’ ability to meet (pay for) care expenses before and after the pandemic began (*P* = .002), with fewer caregivers able to meet most or all care expenses, and more caregivers only able to meet some, very few or no expenses after the pandemic was declared (Figure 1). Caregivers unable to meet all care expenses noted a number of solutions, including modifying or cutting non-care related expenses, increasing reliance on a credit card, using savings, and borrowing money from the bank or family (Supplemental Figure 3).

#### Productivity through formal employment

Roughly half of all caregivers (n = 86; 95% CI 42.3%-57.7%) were not working in the formal (paid) labor market, either because they were retired or unemployed. Among working caregivers (n = 86; 50%; 95% CI 42.3%-57.7%), 77.9% (ns = 67; 95% CI 67.7%-86.1%) reported challenges to productivity due to caregiving (not necessarily attributable to the pandemic), including having to modify their working schedule (start late, leave early, or take time off during the day) to perform a care-related activity an average of 13.55 times (ns = 60; SD 15.450; 95% CI 9.56-17.54) over the past 3 months. Working caregivers also reported changes to retirement timing, with 31.3% (ns = 25; 95% CI 21.3-42.6) planning to retire later due to caregiving, and 8.8% (ns = 7; 95% CI 3.6%-17.2%) planning to retire sooner.

**Figure 2.**
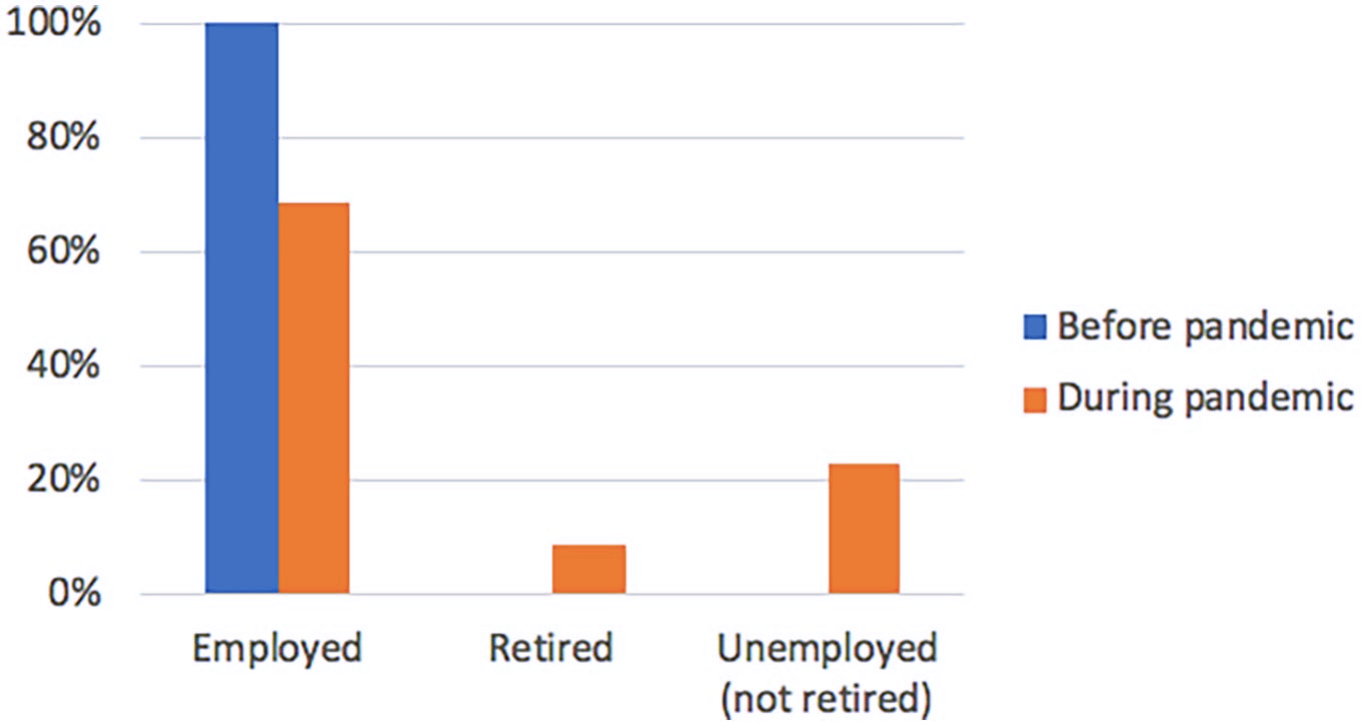
Change in employment status before & after COVID-19 pandemic declared (n = 39).

55.2% of all caregivers (n = 101; 95% CI 47.4%-62.5%) reported a change to their employment status in the past that they attributed directly to the onset of caregiving activities, 34.7% (ns = 33; 95% CI 25.3%-45.2%) of whom experienced this change after the pandemic was declared. Changes included retiring voluntarily or involuntarily (forced into early retirement), losing their job (voluntarily quitting or being let go), or experiencing a reduction in weekly hours worked, resulting in a loss of workplace health benefits (Supplemental Figure 4). A binomial test shows a statistically significant difference in employment status before and after the pandemic was declared (*P* = .013), with fewer caregivers employed in the formal labor market and more caregivers retiring or becoming unemployed after the pandemic was declared (Figure 2).

#### Personal health

Although caregivers’ self-rated physical health was high, with 60.5% (n = 115; 95% CI 53.2%-67.5%) reporting good, very good, or excellent physical health, fewer caregivers (n = 89; 46.8%; 95% CI 39.8%-54.5%) reported their mental health as being good or better, attributable to feelings of stress, anxiety or depression due to the financial aspects of caregiving (χ2 = 43.72, *P* = .000, df = 4, φ = 0.48) noted by 39.5% (n = 75; 95% CI 32.5%-46.8%) of all caregivers.

Over half (n = 102; 53.7%; 95% CI 46.3%-60.9%) of all caregivers previously sought mental health care, broadly defined as counseling and/or medication, motivated by caregiving. Of this group, 55.9% (ns = 57; 95% CI 45.7%-65.7%) sought this care in the past 3 months. The average out-of-pocket cost for mental health care was $533.33 CAD (SD $629.67; 95% CI $298.2-$768.5) over the previous 3 months. Among those who never sought mental health care (n = 88), 50% (ns = 44; 95% CI 39.1%-60.9%) noted never needing it, 29.5% (ns = 26; 95% CI 20.3%-40.2%) noted not having the time, and 12.5% (ns = 11; 95% CI 6.4%-21.3%) noted the prohibitive cost.

A sizable group of caregivers (n = 59; 31.1%; 95% CI 24.7-38.3) sought respite sometime in the past. Of this group, 27.1% (ns = 16; 95% CI 16.4%-40.3%) sought respite in the past 3 months, incurring an average out-of-pocket cost of $840.91 CAD (SD $735.47, 95% CI $346.8-$1335). Among those who have never sought respite (n = 131), 43.8% (ns = 57; 95% CI 35.2%-52.8%) noted never needing it; 20.8% (ns = 27; 95% CI 14.2%-28.8%) noted concerns around COVID-19 transmission, and 18.5% (ns = 24; 95% CI 12.2%-26.2%) noted its prohibitive cost.

When asked how caregiving could be made easier to better maintain overall personal health, the majority of caregivers (n = 122; 64.2%; 95% CI 57%-71%) reported the need for public financial assistance to cover direct care-related expenses. Other desires included better physical health and stamina, more hours of publicly subsidized home care provided by a home care worker, access to mental health care, and support from family (Supplemental Figure 5).

## Discussion

Results from this study reveal several patterns concerning the financial risks of homebased caregiving during the COVID-19 pandemic. In this case, financial risk represents both the magnitude of direct (namely out-of-pocket) care expenditure and their consequences across income, employment productivity and personal health. Specifically, caregivers incurred several expenses out-of-pocket for transportation, non-prescription medications, and COVID-19 personal protective equipment. Among the many consequences across the domains of financial risk, 4 stand out: (1) caregivers experienced an increase in weekly total care expenditure during the pandemic, thereby having to modify daily spending behaviors to meet these expenses; (2) work productivity was compromised as a result of taking time off to provide care, with caregivers retiring, reducing hours, or stopping work altogether; (3) there was a significant relationship between deficits in the availability of publicly subsidized hours of personal support work during the pandemic and caregivers, largely of those living with dementia, having to pay out-of-pocket to fill these deficits; and (4) the financial stressors of caregiving during the pandemic have contributed negatively to caregivers’ mental health, which could be partly offset by greater public and employment-based assistance in managing care expenses and productivity losses.

Previous Canadian studies exploring the private costs of caregiving focus largely on monetizing time.^39-41^ The bias toward such studies may be rooted in challenges collecting self-reported estimates of care expenditure, particularly co-payments for medications.^42^ While we do not monetize time in this study, our findings corroborate the influence of time spent caregiving on reduced workforce participation. In addition, more time was spent caregiving during the pandemic than before it, resulting in longer departure from the paid workforce among working caregivers.

While research on self-reported, out-of-pocket caregiving expenditure in Canada is growing, it has focused on specific health conditions, including caregiving for octogenarians in intensive care,^43^ palliative care patients,^39,44^ persons living with Alzheimer’s Disease or related dementia,^45^ children with traumatic brain injury^46^ and persons with intellectual developmental conditions.^47^ Further, these studies report total out-of-pocket caregiving expenditure, and few seek to understand estimates of expenditure across specific cost categories. Even fewer explicitly report how these costs translate into any patterns of financial risk. Existing scholarship may also be outdated as a result of the pandemic, which we show has had important implications on the financial security of caregivers. Thus, findings from our study are unique in that they fill important research gaps concerning the current state of care expenditure and financial risks of caregiving among a large subset of caregivers—those providing care within the home in the context of the pandemic.

Our findings complement recent commentaries about caregiver financial risk in light of the pandemic and the sustainability of long-term care in Canada. Given the rise in home care service provision, Flood et al^48^ identify the need for cash-for-care benefits, or direct public transfers for care recipients or their unpaid caregivers to support care at home. This scheme exists in over half of all OECD countries. Compensating unpaid caregivers’ time may undermine their desire to provide care for a loved one due to a familial obligation (eg, filial piety) and may blur the lines between the role of paid home care workers and unpaid caregivers.^49^ But, it may also give caregivers more control over how their care is organized and provided, akin to the Medicaid consumer-directed care programs in the United States.^48,50^ From a policy perspective, the argument that the economic effects of the aging population could dismantle the welfare state may be flawed; there is immense benefit in public financial investment in the health of older adults—who represent the majority of care recipients in our sample—and correspondingly, their caregivers.^51^

Indeed, the pandemic has yielded some policy responses at the provincial and federal levels, including a provincial worker income protection benefit (paid infectious disease emergency leave)^52^ and the Canada Recovery Caregiving Benefit for working caregivers valued at $500 (taxable) per week for a maximum of 42 weeks.^53^ However, these measures are temporary, and findings from this study suggest that that some caregivers will still be left behind, particularly retired/unemployed caregivers who rely on savings to pay for care expenses.

To our knowledge, this is the first exploratory study of its kind to describe the financial risks of caregiving across a broad sample of unpaid, homebased caregivers during the pandemic and in the Canadian context. While these results focus specifically on Ontario, findings may be generalizable across other Canadian jurisdictions where home care services are not fully publicly funded. Results may also inform the development of similar studies across other high-income federations with publicly funded health care where caregivers provide the majority of home care services.

As we aimed to be descriptive, future studies with a larger sample size should explore the extent to which financial risk is disproportionately experienced across caregiver groups, for example, by caregivers of persons living with specific health conditions, by caregivers from specific sociodemographic backgrounds, and by caregivers across age-categories or employment status. Further, qualitative investigation is needed to elucidate how these impacts are experienced by caregivers and how domains of financial risk interact so that targeted solutions are not siloed across specific domains of financial risk. This research may also inspire research on other dimensions of care costs, including expenses that may linger after a care recipient passes on.

## Limitations

Our sample size may be considered small. This could be due to a combination of feasibility constraints, the sensitive subject-matter of the survey, and pandemic-related limitations around in-person recruitment and conducting paper surveys. Despite these limitations, our sample size is consistent with other caregiving studies using self-reported, cross-sectional surveys in other Canadian contexts.^43,54^ Further, our sample is not wholly representative of the ethnocultural heterogeneity of caregivers in Ontario, reasons for which we speculate elsewhere.^55^ Specifically, our sample represents caregivers who spoke English, had the time to complete an online survey, had access to internet, and self-identified as a “caregiver” per recruitment material. Accordingly, our findings may underestimate the true financial burden of caregiving across the diverse spectrum of caregivers in Ontario.

Additionally, our survey was conducted over 3 months after the pandemic was declared in Ontario to avoid conflating pre-pandemic and pandemic-related estimates of care-related expenditure; however, this means the recall period may not capture high, upfront and one-time expenses incurred at the start pandemic. As with all self-reported surveys involving estimated expenses, there is also a possibility that out-of-pocket estimates were underreported. Lastly, this survey captures income sources beyond individual earnings (ie, wages and salary) including contributions from other household members; however, use of lay terminology such as “personal” income (instead of “household” income) may give a skewed impression of caregivers’ economic status. Findings from the pilot test of this survey are useful in informing larger scale studies that focus more wholly on household income and the financial risks of unpaid caregiving.

## Conclusion

Findings from this study will be important in COVID-19 pandemic recovery efforts both regionally and nationally. The pandemic has exacerbated challenges in caregiving, including the financial challenges. Inevitably, the pandemic will shape the setting in which care is received, and in the process, by whom this care is provided. Without a fulsome understanding of the costs of caregiving borne by caregivers and the corresponding financial risks, policies and programs that aim to protect unpaid caregivers from the financial risks of caregiving will be ill-informed and inconsistent with the overarching ideologies around welfare provision that have long protected users of health care and, by extension, their caregivers.

## Supplemental Material

sj-docx-1-his-10.1177_11786329221144889 – Supplemental material for The Financial Risks of Unpaid Caregiving During the COVID-19 Pandemic: Results From a Self-reported Survey in a Canadian Jurisdiction Click here for additional data file. Supplemental material, sj-docx-1-his-10.1177_11786329221144889 for The Financial Risks of Unpaid Caregiving During the COVID-19 Pandemic: Results From a Self-reported Survey in a Canadian Jurisdiction by Husayn Marani, Sara Allin, Sandra McKay and Gregory P. Marchildon in Health Services Insights
