# Supplementary material for: The Financial Risks of Unpaid Caregiving During the COVID-19 Pandemic: Results From a Self-reported Survey in a Canadian Jurisdiction
Source: Health Serv Insights. 2023 Jan 7;16:11786329221144889. doi: 10.1177/11786329221144889 (PMC9827143; doi:10.1177/11786329221144889)
Supplement: sj-docx-1-his-10.1177_11786329221144889 – Supplemental material for The Financial Risks of Unpaid Caregiving During the COVID-19 Pandemic: Results From a Self-reported Survey in a Canadian Jurisdiction [file sj-docx-1-his-10.1177_11786329221144889.docx]

**ONLINE SUPPLEMENT**

**
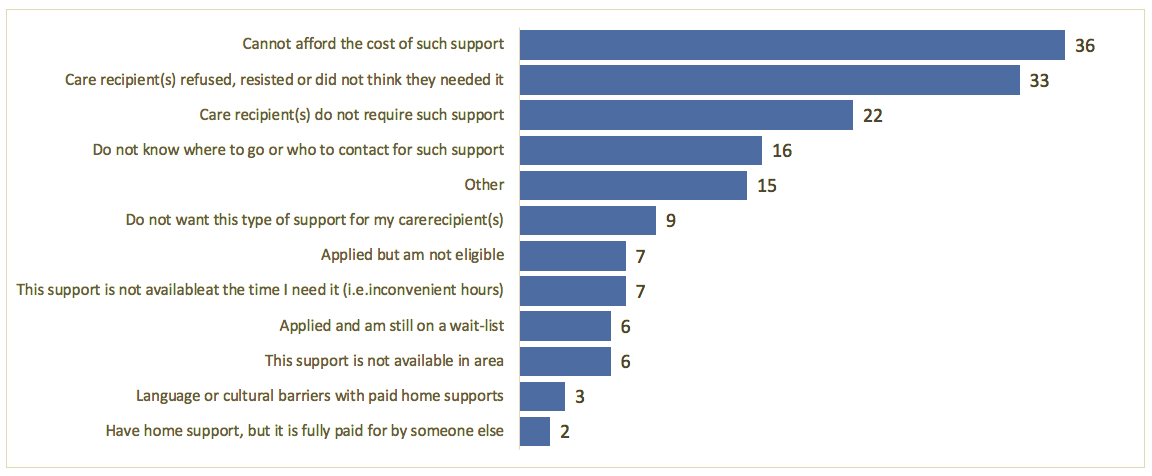
**

**Supplement Figure 1** Reasons for not paying for supplemental support from a home care worker (percent of those not receiving home care from a home care worker, n=100)

**
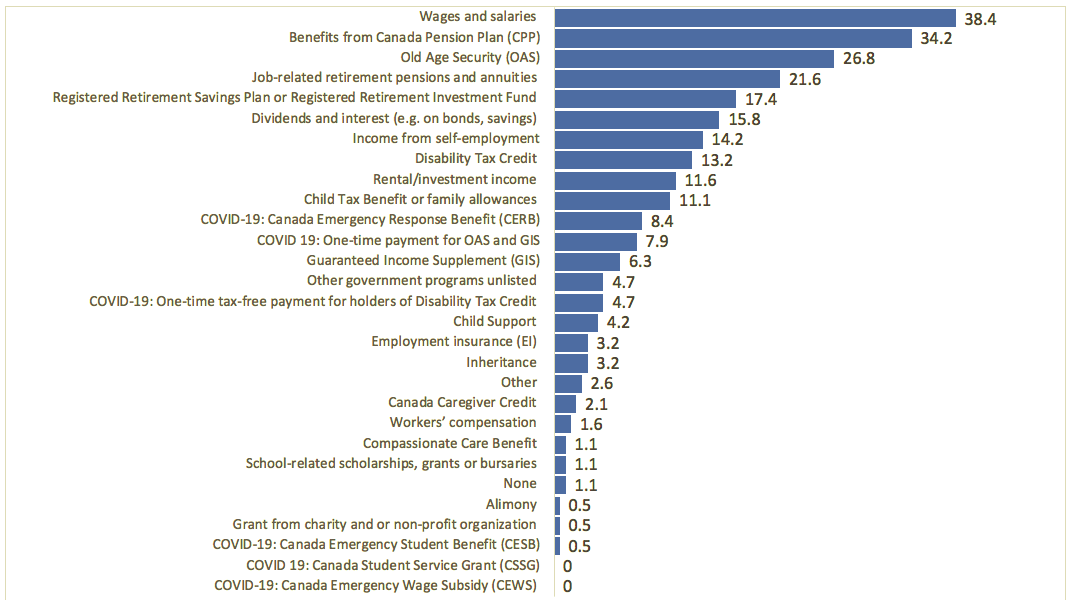
**

**Supplement Figure 2** Sources of income in the past three months (percent of full sample, n=190)

**
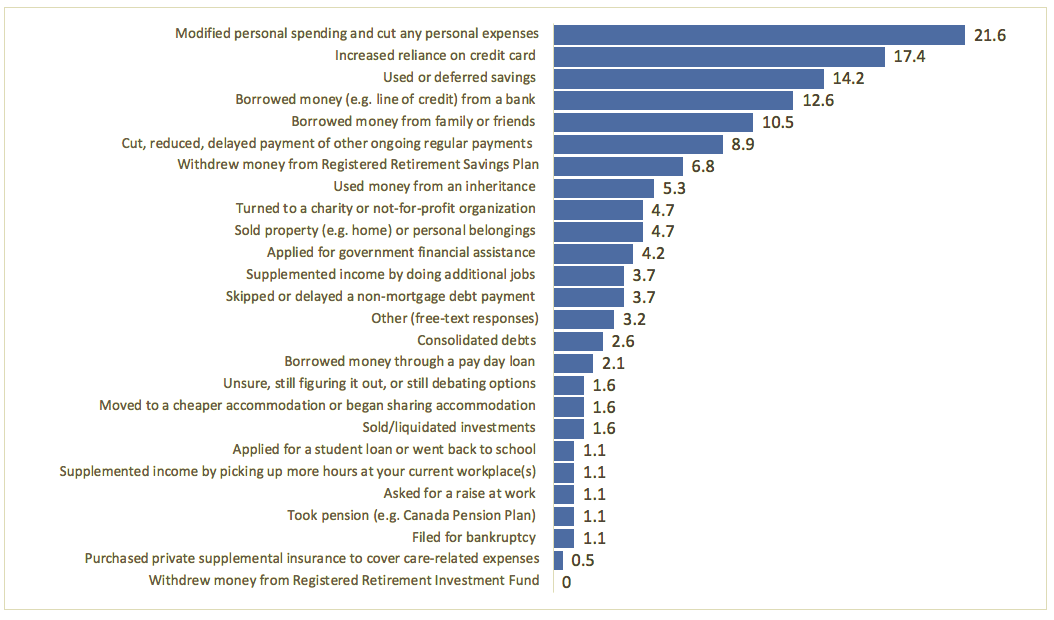
**

**Supplement Figure 3** Strategies to meet care expenses over caregiving history (percent of those who could not meet all care expenses, n=75)

**
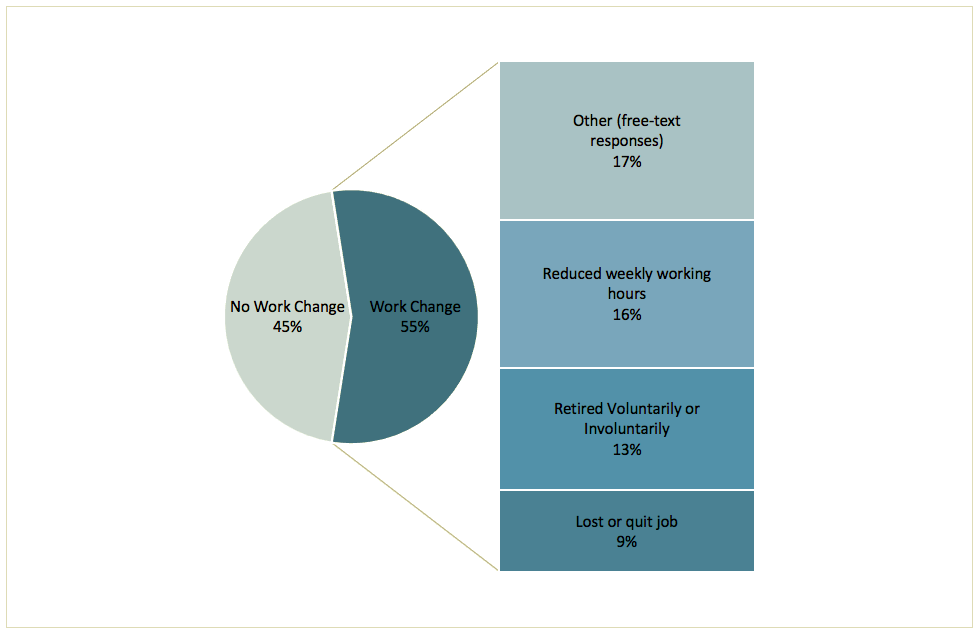
**

**Supplement Figure 4** Change in employment status due to caregiving (percent of full sample, n=190)

**
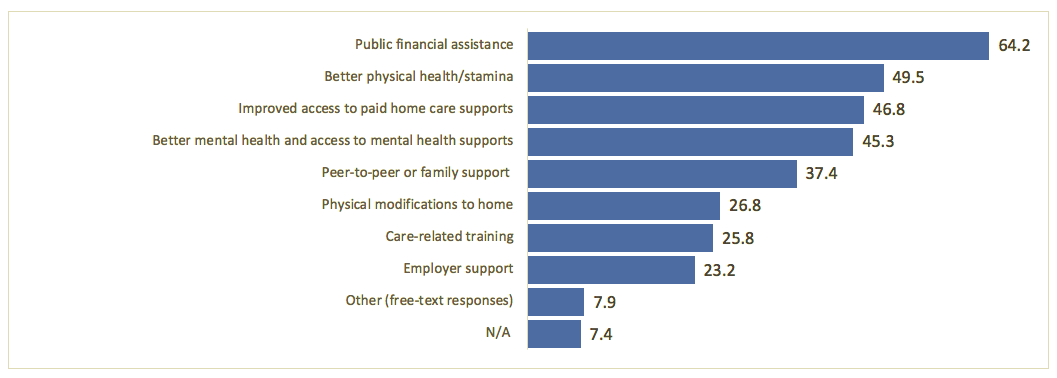
**

**Supplement Figure 5** Desired supports to sustain or improve health of caregivers (percent of full sample, n=190)
